# Associations between smokers’ knowledge of causes of smoking harm and related beliefs and behaviors: Findings from the International Tobacco Control (ITC) Four Country Smoking and Vaping Survey

**DOI:** 10.1371/journal.pone.0292856

**Published:** 2023-10-24

**Authors:** Bill King, Ron Borland, Michael Le Grande, Destiny Diaz, Richard O’Connor, Katherine East, Eve Taylor, Coral Gartner, Hua-Hie Yong

**Affiliations:** 1 Melbourne Centre for Behaviour Change, School of Psychological Sciences, The University of Melbourne, Parkville, Victoria, Australia; 2 Roswell Park Comprehensive Cancer Center, Elm and Carlton Streets, Buffalo, NY, United States of America; 3 Department of Addictions, Institute of Psychiatry, Psychology and Neuroscience, National Addiction Centre, Kings College, London, United Kingdom; 4 Faculty of Medicine, School of Public Health, The University of Queensland, Herston, QLD, Australia; 5 School of Psychology, Deakin University, Geelong, Victoria, Australia; UV: Universiteti Ismail Qemali Vlore, ALBANIA

## Abstract

**Background:**

Most smokers know that smoking is harmful to health, but less is known about their understanding of what causes the harms. The primary aim was to examine smokers’ perceptions of the relative contributions to smoking-related morbidity from combustion products, nicotine, other substances present in unburned tobacco, and additives. A secondary aim was to evaluate the association of these perceptions with nicotine vaping product use intentions, and quitting motivation/intentions.

**Methods:**

Participants were current smokers and recent ex-smokers from Australia, Canada, England and the United States (N = 12,904, including 8511 daily smokers), surveyed in the 2018 International Tobacco Control Four Country Smoking and Vaping Survey. Respondents reported on how much they thought combustion products, nicotine, chemicals in the tobacco and additives in cigarettes contribute to smoking-related morbidity (none/very little; some but less than half; around half; more than half; all or nearly all of it; don’t know).

**Results:**

Overall, 4% of participants provided estimates for all four component causes that fell within the ranges classified correct, with younger respondents and those from England most likely to be correct. Respondents who rated combustion as clearly more important than nicotine in causing harm (25%) were the least likely to be smoking daily and more likely to have quit and/or to be vaping. Among daily smokers, all four cause estimates were independently related to overall health worry and extent of wanting to quit, but the relative rating of combustion compared to nicotine did not add to prediction. Those who answered ‘don’t know’ to the sources of harm questions and those suggesting very little harm were consistently least interested in quitting.

**Conclusions:**

Most smokers’ knowledge of specific causes of harm is currently inadequate and could impact their informed decision-making ability.

## Introduction

Anti-smoking education campaigns have generally focused on disseminating information about the range and magnitude of smoking-related morbidity/mortality [1]. These efforts are grounded in expectancy value theories (e.g., the Health Beliefs Model), which hold that understanding the severity of a problem, along with a sense of one’s personal susceptibility, will motivate taking protective action [2–4]. This general approach has been effective in motivating smoking cessation and deterring uptake [3,5], perhaps by evoking feelings of concern which motivate the search for potential actions [4,6]. Even so, anti-smoking education campaigns generally have not informed consumers about how smoking causes harm, and while nearly everyone is aware that smoking is harmful to health, most people underestimate the risk to their personal health [7].

Smoking-related harms largely come from prolonged exposure over decades to the toxicants in tobacco smoke, exacerbated by the site of exposure (lungs rather than mouth and/or stomach) [8,9]. A clear dose-response relationship with cumulative exposure to toxicants exists, as estimated by the average number of cigarettes smoked per day [10,11]. Better understanding of the sources of smoking-related toxicant exposures may facilitate better choices about use of cigarettes or other nicotine-containing products.

The toxicants produced from smoking are predominantly by-products of the partial combustion of the tobacco (e.g., polyaromatic hydrocarbons, volatile organic compounds, carbon monoxide) [12,13], plus toxicants present in the unburnt tobacco that are transferred to the smoke [9,14]. Among the products coming directly from tobacco, nicotine *per se* has low toxicity at the levels found in smoking. However, as nicotine is the main reason people smoke, it is important to separate its potential contribution to harm from that of other substances. We accept that nicotine indirectly contributes to smoking-related harm as dependance leads to prolonged exposures.

Recent research on knowledge of the causes of smoking-related harm has focused on four component causes listed in Box 1.

**Box 1. Contributors to harms from smoking**

**Table:** 

**Component cause of harm**	**Estimate of contribution**
Combustion: Tobacco combustion products	Well over half of the risk [12,13]. Combustion creates many of the toxicants.
Tobacco: Toxic substances in unburnt tobacco	Some, but well under half, the risk [9,14].
Additives; e.g., flavorings and humectants	A small fraction of the total risk [13,15]. They constitute a small fraction of a cigarette’s weight and thus little added, even when partly combusted.
Nicotine	Contributes little to the risk directly [9,14,16,17]; (i.e., excluding indirect effects due to dependance).

Smokers appear to be poorly informed about these sources of harm [18–20]. Only a minority believe that combustion products are the main source of tobacco-related harm. Further, many believe that either additives or nicotine are the main sources of harm. It has also been shown that smokers who replied “don’t know” to questions about component causes had particularly low interest in quitting suggesting disengagement from thinking about smoking harms [19].

Misperceptions of nicotine harms relative to combustion [21–23] may contribute to underuse of effective aids (e.g., Nicotine Replacement therapy, NRT) or undermine consideration of switching to less harmful nicotine products (e.g., nicotine vaping products (NVPs), low-toxicant oral tobacco). They may also be used to justify common misbeliefs that ‘natural,’ ‘additive free,’ and roll-your-own cigarettes are lower in risk, and thus potential alternatives to quitting [18,19] The magnitude of harm reduction short of quitting all nicotine products is likely a function of the extent of toxicant reduction and the mode of use (oral versus lung inhalation).

The present study extends our prior work [19], replicating and extending it on larger and more representative samples of smokers from four broadly comparable countries. The primary aim is to examine smokers’ perceptions of the relative contributions to smoking-related morbidity from combustion products, nicotine, other substances present in unburned tobacco, and additives.

A secondary aim was to evaluate whether individual measures of sources of smoking-related harm, and measures of relative concern, were associated with greater interest in quitting smoking and use of other nicotine products as would be predicted from an understanding-based model of quitting (i.e., deeper understanding drives increased motivation), while a more affect-based model (ie emotional concern is the driver) would predict higher concern to be predictive independent of the validity of the concern, with relative concern potentially less important. Finally, we sought to confirm if smokers who claim they “don’t know” about the relative harms are particularly resistant to thinking about quitting and can reasonably be described as “disengaged”.

### Methods

#### Data sources

The sample is from the 2018 wave of the International Tobacco Control (ITC) Four Country Vaping and Smoking Survey, an online cohort survey conducted in Canada, United States, England and Australia. It replenishes using the same sampling frame as for recruitment and is structured to provide a representative sample of the respective population within each country. The data reported here come for the third wave of the survey. Respondents (adults ≥ 18 years) were recruited by commercial panel firms in each country as any of established cigarette smokers (smoke at least monthly, and smoked at least 100 cigarettes in their lifetime), recent ex-smokers (quit ≤ 2 years), or vapers (vape ≥ weekly) and all were retained where possible. The resultant samples were weighted to be representative of the appropriate population within each country. Full descriptions of the conceptual framework [24] and detailed sampling methods [25] have been published. The current analysis includes all current smokers (daily and non-daily) and ex-smokers (quit for less than 4 years), totaling 12,904 cases (Canada: n = 3734, United States: n = 2810, England: n = 4846 and Australia: n = 1514). Multivariate analysis was restricted to daily smokers (n = 8511).

#### Ethics approval

Study questionnaires and materials were reviewed and provided clearance by Research Ethics Committees at the following institutions: University of Waterloo (Canada, ORE#20803/30570, ORE#21609/30878), King’s College London, UK (RESCM-17/18–2240), Cancer Council Victoria, Australia (HREC1603), Deakin University, Australia (HREC2018-346), University of Queensland, Australia (2016000330/HREC1603); and Medical University of South Carolina (waived due to minimal risk). All participants provided written consent to participate.

#### Measures

Basic demographic data included country of residence (Canada, United States, England, Australia), gender, age (18–24, 25–39, 40–54, 55+). As indices of socio-economic status, we used education (“low” if completed high school or less in Australia, Canada, and the US, or secondary or less in the UK, “medium” if completed college/university (no degree) in the UK, technical/trade/some university (no degree) in Australia, or community college/trade/technical school/some university (no degree) in Canada and the US, or “high” if completed university or postgraduate in all countries) and reported financial stress [26] assessed by:” In the last 30 days, because of a shortage of money, were you unable to pay any important bills on time, such as electricity, telephone or rent bills?” (Yes, No/Don’t know).

Current smoking status was divided into three categories: current daily, current non-daily (less than daily but at least monthly), and recent ex-smokers (within 4 years), by asking ever smokers: “How often do you CURRENTLY smoke ordinary cigarettes (either factory-made/pack or roll-your-own)?” with response options including: Daily; Less than daily, but at least once a week; Less than weekly, but at least once a month; and I have quit smoking.

Nicotine vaping product use was assessed as daily, less than daily (but at least monthly), past regular use, and never regularly vaped or never vaped at all.

#### Perception of component causes of harm

Following King et al, 2019 [19], we asked: ‘How much of the disease caused by cigarette smoking comes from the following? (a) the nicotine in tobacco; (b) other harmful substances that occur naturally in unburnt tobacco; (c) harmful substances that are produced when the tobacco burns; and (d) substances that are added to cigarettes during the manufacturing process’, presented in this order. Response options for each item were: (i) none or very little; (ii) some but less than half; (iii) around half; (iv) more than half; (v) all or nearly all of it; and (vi) Don’t know. These roles are referred to from here as Nicotine, Tobacco Combustion and Additives respectively. Responses classified as correct were (i) and (ii) for both Nicotine and Additives roles; (i), (ii) and (iii), for unburnt Tobacco; (iv) and (v) for Combustion. All responses classified as incorrect were underestimates of the role of combustion, but overestimates for Tobacco, Nicotine, and Additives.

We also used questions that were asked about nicotine and vaping:

For ‘Relative NVP harm’, we asked: “Compared to smoking cigarettes, how harmful do you think vaping (using e-cigarettes) is?” Response options were: Much less; Somewhat less: Equally; somewhat more; Much more, with ‘harmful than smoking cigarettes’ added to each option; plus a ‘Don’t know’ option.

For ‘Relative NVP addiction’, we asked a parallel question: “Compared to smoking cigarettes, how addictive do you think vaping (using e-cigarettes) with nicotine is?” With the same response options, with ‘addictive’ replacing ‘harmful’ for each option; plus ‘Don’t know’.

For “Worry”, we asked: “How worried are you, if at all, that smoking cigarettes WILL damage your health in the future?” with response options: 1) Not at all worried, 2) A little worried, 3) Moderately worried; 4) Very worried; and ‘Don’t know’.

For “Want to quit”, we asked: “How much do you want to quit smoking?” with response options: 1) Not at all, 2) A little, 3) Somewhat, 4) A lot, and ‘Don’t know’.

For “Plan to quit”, all current smokers were asked: “Are you planning to quit smoking: (4) Within the next month; 3) Between 1–6 months from now; 2) Sometime in the future beyond 6 months; or (1) Not planning to quit; plus ‘Don’t Know’ which was combined with ‘Not planning’ and labelled ‘No plans’.

For “Future NVP use”, those not currently vaping were asked about future likelihood of vaping with the question: “How likely are you to use e-cigarettes or e-liquids that CONTAIN NICOTINE in the future? (This means more than just trying them),” with response options: 5 Definitely will use; 4 Probably will use; 3 Might or might not use; 2 Probably will not use; 1 Definitely will not use; and ‘Don’t Know’.

#### Composite measures

To assess whether risk estimates of the four components of smoking-related harm serve as probability estimates, we summed the score for all 4 (but excluding any with ‘Don’t know’ responses) treating ‘Little or none’ as 0.0; ‘Some but less than half’ as 0.2, ‘About half’ (0.4), ‘More than half’ (0.6), and ‘All or nearly all’ (0.8), i.e., the estimate was set at the lower bound of each likely range when the range is divided into five parts (i.e., 0 for 0–0.2 for ‘Little or none’).

For “Relative concern”, we created a 5 segment measure, modifying the previously used measure [19], from the “Nicotine and Combustion” component questions: (1, combustion products rated two or more points higher than nicotine on the 5-point response scales (Combustion most harm); 2, combustion rated one point higher than nicotine (Combustion just); 3, combustion and nicotine rated equally (non-discriminating); 4, nicotine rated higher than combustion (Nicotine most); and 5, ‘Don’t know’: either to both, or Don’t know to nicotine and half or less for “Combustion’s” role as half or more of the harm was not “accounted for”. In some analyses, the three middle segments (i.e., Combustion just, Non-discriminating and Nicotine most) were described as ‘Inaccurate’, as they all underestimate the role of combustion relative to nicotine.

See S1 Table for an outline of the key measures of harm perception and where they appear in the results.

#### Data analysis

All data analyses were conducted using Stata V16.1 (StataCorp, TX). We used simple descriptives and for country specific estimates, report country weighted data, but otherwise analyzed unweighted data, controlling for other variables where indicated. The use of analytical weights was not necessary for our associations of interest, and were by-country, a measure not of focal interest here, and may produce less variability and standard errors than weighted data [27]. We calculated two sets of inferential statistics using Chi-Square analysis for examining the relationships between the segments of the relative concern measure and other categorical variables. We corrected for multiple comparisons using the Benjamini-Hochberg method [28] We compared the “Disengaged” segment and all others, plus between “Combustion most” and the 3 inaccurate segments. This was complemented by ordinal logistic regressions treating the other variables we were interested in as the outcomes (future NVP use, Want to quit, Plan to quit, and Worry), which required dropping any “Don’t know” responses from these measures, to make them ordinal. All regressions were adjusted for age, gender, country, level of education, financial stress and vaping status. A second set of models also controlled for perceived harm and addictiveness of NVP compared to smoking, plus Worry where appropriate.

The analysis was not pre-registered and the results should be considered exploratory.

## Results

Table 1 presents a breakdown of the sample by smoking status. Most participants (66.0%) smoked daily, with 14% smoking non-daily and 20% recently quit. Among daily smokers, 18% vaped weekly or monthly and 14% vaped daily.

**Table 1:** Demographics, smoking, vaping and quitting status of the 2018 International Tobacco Control Four Country Smoking and Vaping Survey study sample.

		Total Sample		Daily Smokers	
		N	%	N	%
Variable		12904		8511	
**Country**	Canada United States England Australia	3734 2810 4846 1514	28.9% 21.8% 37.6% 11.7%	2185 1678 3477 1171	25.7% 19.7% 40.9% 13.8%
**Gender**	Female Male	6618 6286	48.7% 51.3%	4141 4370	48.7% 51.3%
**Age**	18–24 25–39 40–54 55+	2506 2838 3316 4244	19.4% 22.0% 25.7% 32.9%	1446 1746 2353 2966	17.0% 20.5% 27.6% 34.8%
**Education**	Low Medium High	4006 5492 3406	31.0% 42.6% 26.4%	2894 3545 2072	34.0% 41.7% 24.3%
**Financial stress**	No Yes	10889 2015	84.4% 15.6%	7044 1467	82.8% 17.2%
**Smoking status**	Daily smoker	8511	66.0%	8511	100%
	Non-daily	1770	13.7%		
	Currently quit smoking	2623	20.3%		
**Vaping status**	Daily vaper Less than daily Not currently vaping	2356 2325 8223	18.3% 18.0% 63.7%	1185 1504 5822	13.9% 17.7% 68.4%

Note: Percentages are unweighted.

Table 2 presents summarized results for the four component cause items by smoking status (response category ranges classified as correct/approximately correct are highlighted). In the total sample and in daily smokers, correct identification of harm ranged from 25% for additives) to 47% for unburned tobacco) with nicotine (36%) and tobacco combustion (42%) in between. Ex-smokers (49%) were significantly more likely to correctly identify combustion as a source of harm compared to daily or non-daily smokers (both close to 40%). To a lesser extent a similar pattern was found for nicotine (41% correct in ex-smokers compared to 35% in daily smokers).

**Table 2:** Percentages of responses in each category and percentages of responses rated correct for the four component cause items. Responses accepted as within the range of correctness are shaded. Data from the 2018 International Tobacco Control Four Country Smoking and Vaping Survey study sample.

	Responses						
Role in causing harm	None or very little	Some but less than half	Around half	More than half	All or nearly all	Don’t know	% in correct range
**Combustion products of tobacco:**							
All smokers	2.4	16.6	19.5	**21.9**	**19.8**	19.8	41.7
Daily smokers	2.7	17.2	19.4	**21.5** ^**a**^	**18.0** ^**a**^	21.3	39.5^a^
Non-daily	2.7	17.2	23.8	**22.0** ^**a**^	**18.6** ^**a**^	15.7	40.6^a^
Recent ex-	1.5	14.3	17.0	**23.0** ^**a**^	**26.4** ^**b**^	17.7	49.4^b^
**Nicotine**							
All smokers	**14.7**	**21.4**	17.3	14.8	13.7	18.2	36.1
Daily smokers	**13.5** ^**a**^	**21.0** ^**a**^	17.5	14.6	14.1	19.3	34.5^a^
Non-daily	**12.8** ^**a**^	**23.8** ^**b**^	21.2	15.4	11.6	15.3	36.6^a^
Recent ex-	**19.7** ^**b**^	**21.4** ^**a**^	13.7	14.8	13.8	16.7	41.3^b^
**Other substances in unburned tobacco**							
All smokers	**5.7**	**21.3**	**20.0**	17.8	14.9	20.4	47.0
Daily smokers	**5.7** ^**a**^	**20.5** ^**a**^	**20.4** ^**a**^	17.6	14.2	21.7	46.6^a^
Non-daily	**5.5** ^**a**^	**23.7** ^**b**^	**22.6** ^**a**^	19.3	12.2	16.7	51.8^b^
Recent ex-	**5.8** ^**a**^	**22.2** ^**b**^	**16.9**_**b**_	17.3	18.9	19.0	44.9^a^
**Additives**							
All smokers	**6.7**	**18.2**	15.9	19.1	18.6	21.5	24.9
Daily smokers	**6.6** ^**a**^	**18.2** ^**b**^	16.0	18.4	17.8	23.0	24.8^a^
Non-daily	**7.3** ^**a**^	**20.2** ^**b**^	19.3	20.0	16.1	17.0	27.5^b^
Recent ex-	**6.8** ^**a**^	**16.9** ^**a**^	13.2	20.6	23.0	19.6	23.7^a^

Figures are row percent; n all smokers = 12,904, daily smokers = 8,511, non-daily = 1,770, recent ex-smokers = 2,623; For correct range only, each subscript denotes a subset of smoking status categories whose cell proportions do not differ significantly from each other after adjusting for multiple comparisons using the Benjamini-Hochberg method.

The remaining analyses focused on daily smokers as they are most affected by understanding of smoking harms. Overall, correct assessment of the roles of the four components of smoking harm was lowest for additives (25% correct), through to nicotine (34%), combustion (39%) and highest for natural tobacco (46% correct). There were significant by-country differences for responses to all four component cause items. Among daily smokers, most variation by country occurred for nicotine’s role, ranging from 40% correct in England, followed by Australia (35%), Canada (31%) to 28% in the US (all adjacent comparisons *p* <0.05). In contrast, there was no significant variation in the understanding of the role of combustion, ranging from 41% correct in Canada through to 38% in the US. Correct assessment of the role of unburnt tobacco was highest in Canada (49%) and England (48%) and significantly lower (*p* <0.05) in the US (45%) and Australia (43%). There were also age effects. For the role of unburnt tobacco, younger smokers (56% correct) had a significantly better understanding than older smokers (41%). Similarly, the youngest age group (40% correct) understood the role of nicotine better than the 55+ age group (32%).

When ‘Don’t Knows’ were excluded, responses to all four items were positively correlated. Among daily smokers this ranged from *r* = 0.12 for nicotine and additives, *r* = 0.15 for combustion and nicotine through to *r* = 0.53 for combustion and natural tobacco (all p <0.001). If the respondents had been estimating approximate probabilities as asked, some items would need to be negatively correlated for the four fractions to add to one (or less, if respondents considered other independent causes). Indeed, using the quantitative estimates described above, when summed, only 25.5% gave responses that added to 1.0 or less. Indeed, 21.6% gave responses that added to more than 2.0. The quantitative estimate was moderately correlated with “Worry ‘about future health’” (excluding “Don’t know” responses) (*r* = 0.23, p < .001) consistent with the judgements being influenced by a generalized concern about smoking harms.

To assess whether ‘Don’t know’ responses were particularly resistant to quitting, we cross-tabulated the combustion role measure, as the only example we have of a clear underestimation of risk, with “Want to quit” and “Plan to quit”. We found that responding ‘Little or no’ role was associated with less wanting and lower plans to quit, but that ‘Don’t know’ came between this and ‘Some but less than half’ responses (see S2 Table).

For the composite 5-segment measure of Relative Concern between combustion and nicotine, only 22.9% of daily smokers were in the ‘Combustion most’ segment of ‘Relative concern’, more than half fitted into the 3 inaccurate segments (57.3%), and 19.8% ended in the ‘Don’t know’ segment, confirming this was a relatively common response pattern (Table 3).

**Table 3:** Relative concern segments by vaping status and vaping relative harm (daily smokers only, n = 8511). Data from the 2018 International Tobacco Control Four Country Smoking and Vaping Survey study sample.

	Relative concern (computed measure)						
	n	11.Combustion most %	2.Combustion just %	3.Non-discriminating %	4.Nicotine most %	5.Don’t know %	Test of group differences
		N = 1952	N = 1001	N = 1795	N = 2079	N = 1684	
**Overall %**		22.9	11.8	21.1	24.4	19.8	
**Vaping status** (row %)							
Daily vaper	1185	24.8	16.9	18.4	29.4	10.6	1–5 X^2^(8) = 198.2, *p* <0.001
Less than daily vaper	1504	26.1	12.8	22.2	25.9	12.9	1 v 2–4 X^2^(2) = 1.7, *p* = 0.417
Non-vaper	5822	21.7	10.4	21.4	23.0	23.5	1–4 v5 X^2^(2) = 157.8, *p* <0.001
**Harm: Vaping v smoking (column percentages)**							
Much less harmful	1432	28.1	16.1	14.6	14.0	10.9	1–5 X^2^(16) 1203.3, *p* < .001 1 v 2–4 X^2^(4) = 273.3 *p*<0.001 1–4 v5 X^2^(4) = 857.2*p* < .001
Somewhat less	3036	42.7	38.1	38.8	35.1	25.1	
Equally harmful	2023	15.8	28.7	25.8	31.4	20.3	
More harmful	545	4.5	9.4	6.5	9.2	3.6	
Don’t know	1404	8.9	7.8	14.4	10.3	40.1	

The Relative concern measure was strongly associated with NVP relative harm, demonstrating some construct validity (See Table 3). However, the relationship was non-linear, with ‘Combustion most’ segment strongly associated with NVPs perceived to be low in harm compared to cigarette smoking. The relationships were weaker than would be expected from an integrated set of beliefs, with some conceptually inconsistent responses. Believing combustion causes more harm than nicotine is inconsistent with believing vaping is more harmful than smoking, yet among those in the ‘Combustion most’ segment, 4.5% believed vaping is more harmful than smoking and another 15.8% thought vaping is equally harmful. It is notable that the ‘Don’t know’ group were much more likely to also provide ‘Don’t know’ responses to other belief items, while there were negligible numbers of such responses to non-belief related items, suggesting genuine uncertainty about these items rather than an overall tendency to respond as ‘Don’t know’.

To explore relationships between the four component cause measures and whether they individually or together relate to NVP interest and three quit-related measures (Worry, Want and Plan), we converted the responses to each component cause measure into three levels (accurate, inaccurate and don’t know) using the response categories in Table 2.

Using ordinal logistic regressions (see Table 4), we explored the relationship between the four 3-level component cause measures and four outcomes: worry smoking will damage health, wanting to quit, planning to quit, and interest in using NVP in the future (not shown). In the first set of models (Table 4A) we controlled for demographics and then added the Relative concern measure (only including it in the final model if it added predictive power). As these measures did not contribute predictive power for NVP interest apart from ‘nicotine most’ with the Relative concern measure being negatively associated with interest (*OR* = .70, 95% CI = 0.54–0.91, *p* < .01) these results are not shown in table. For the three quit-related measures, accurate perceptions of Combustion was associated with greater odds of worry that smoking will harm health and greater desire to quit but there was no relationship with planning to quit. Accurate perceptions of Tobacco, Nicotine, and Additives roles were all negatively associated with the three quit outcomes in comparison to those with inaccurate perceptions. Relative concern also added significant predictive power. For all three quit outcomes, the reference category for Combustion ‘combustion clear top’ was associated with the highest odds for all three outcomes, although not all differences were significant.

**Table 4:** Ordinal logistic regression model using role-related measures to predict outcomes of interest. Data from the 2018 International Tobacco Control Four Country Smoking and Vaping Survey study sample.

	Worry smoking will damage health		Want to quit		Plan to quit	
***A*. *Multivariable analyses including the four role measures and Relative concern***						
**Role measure#**	N = 8280		N = 8511		N = 8511	
**Combustion**	OR sig	95% CI	OR sig	95% CI	OR sig	95% CI
Inaccurate	Ref		Ref		Ref	
Accurate	**1.57** ***	1.36,1.81	**1.43** ***	1.25,1.65	1.04	0.91,1.20
Don’t Know	**1.34** **	1.10,1.65	1.08	0.88,1.32	0.90	0.74,1.10
**Nicotine**						
Inaccurate	Ref		Ref		Ref	
Accurate	**0.62** ***	0.53,0.71	**0.67** ***	0.58,0.77	**0.82** **	0.72,0.95
Don’t Know	0.83	0.66,1.03	0.80*	0.65,0.98	0.86	0.70.1.07
**Tobacco**						
Inaccurate	Ref		Ref		Ref	
Accurate	**0.66** ***	0.60,0.73	**0.70** ***	0.63,0.77	**0.77** ***	0.70,0.86
Don’t Know	**0.69** ***	0.58,0.82	**0.79** **	0.67,0.93	**0.78** **	0.66,0.92
**Additives**						
Inaccurate	Ref		Ref		Ref	
Accurate	**0.78** ***	0.71,0.87	**0.78** ***	0.71,0.86	**0.83** ***	0.75,0.92
Don’t Know	0.85*	0.74,0.97	**0.74** ***	0.65,0.84	**0.75** ***	0.66,0.86
**Relative concern**						
Combustion clearly top	Ref		Ref		Ref	
Combustion just	**0.76** **	0.64,0.90	0.88	0.75,1.05	0.93	0.79,1.10
Equal	0.81	0.64,1.03	0.90	0.71.1.04	**0.79** *	0.63,0.99
Nicotine most	**0.74** **	0.62,0.89	**0.83** *	0.69,0.99	**0.78** **	0.66,0.93
Don’t Know	**0.52** ***	0.40,0.69	**0.77** *	0.59,1.00	**0.65** **	0.50,0.85
***B*. *Multivariable models including other variables listed below ###***						
**Role measures**	N = 8211		N = 8395		N = 8395	
**Combustion**						
Inaccurate	Ref		Ref		Ref	
Accurate	**1.76** ***	1.59,1.95	**1.19** **	1.08,1.32	1.00	0.91,1.11
Don’t Know	1.08	0.91,1.27	1.04	0.88,1.23	**0.82** *	0.70,0.97
**Nicotine**						
Inaccurate	Ref		Ref		Ref	
Accurate	**0.63** ***	0.57,0.69	**0.79** ***	0.72,0.87	1.02	0.93.1.12
Don’t Know	**0.71** ***	0.61,0.83	0.92	0.79.1.08	0.94	0.81,1.10
**Tobacco**						
Inaccurate	Ref		Ref		Ref	
Accurate	**0.67** ***	0.61,0.75	**0.81** ***	0.73,0.90	**0.89** *	0.80,0.98
Don’t Know	**0.71** ***	0.60,0.84	0.92	0.77,1.10	0.90	0.76,1.06
**Additives**						
Inaccurate	Ref		Ref		Ref	
Accurate	**0.77** ***	0.69,0.85	**0.83** ***	0.75,0.91	**0.87** **	0.79,0.96
Don’t Know	**0.85** *	0.74,0.97	**0.79** ***	0.69,0.90	**0.82** **	0.72,0.94

* p < 0.05

** p < 0.01

*** < 0.001.

#Models in 4A include all four role measures and relative concern adjusted for socio-demographics (age, gender, country, education, financial stress, ethnicity) and vaping status.

## Models in 4B include all measures in 4A relative addictiveness of vaping compared to smoking, relative harm of vaping compared to smoking, and for Want and Plan only, worry smoking will damage health in the future.

In the second set of models (Table 4B) we added the NVP relative harm and addiction measures, and for Want to quit and Plan to quit, also added Worry. In all cases, the NVP relative addiction variable was not significant, its bivariate relationship swamped by NVP relative harm. Adding the NVP relative harm measure eliminated a role for Relative concern, but there were continuing predictive effects for some of the four component cause measures for the three quit-related measures. We found that higher concern on all four individual component cause measures was associated with greater Worry and Want, but less so for Plan where only ‘Tobacco’ and ‘Additives’ were significant. In all cases, there were some significant effects with ‘Don’t Know’ being associated with less interest in quitting.

## Discussion

This study confirms previous findings that the vast majority of smokers and recent ex-smokers have inaccurate perceptions of the relative contributions of the four component causes of harm from cigarette smoking (chemicals generated by combustion, chemicals naturally in unburnt tobacco, nicotine, and additives). Fewer than half held correct perceptions of each of the four sources individually, with the contribution of additives most likely to be overestimated. Daily smokers were more likely to have misperceptions of sources of smoking-related harm than non-daily smokers and recent ex-smokers. Fewer than 4% of respondents provided responses that were consistent with a quantitative understanding of the relative risks. Further, respondents clearly did not respond in terms of relative harm when making individual component cause judgements because most estimates did not summed to more than 1.0, the maximum for a probabilistic risk assessment. We also found frequent inconsistencies in response to related knowledge/belief items, even among those who believed that combustion caused the most harm, suggesting this knowledge is poorly integrated.

The findings do not support our second hypothesis that respondents prone to answering ‘Don’t know’ to knowledge related questions will be most disengaged from thinking about quitting. Those who trivialized the large risk from combustion were least engaged.

The finding that English respondents were most likely to have more evidence-consistent beliefs about contributors to harm than in the other three countries may be a function of the recent public education efforts concerning nicotine products as less harmful alternatives to smoking. However, even amongst English smokers, understanding was low.

Answers to the relative risk of NVP use questions show that smokers can make relative comparisons when forced, albeit often inaccurately, with a lot still accepting they ‘Don’t Know’. These measures replaced the small independent effect of our Relative Concern measure. This suggests we should use forced choice measures to assess relative concerns. Given the NVP relative harm measure assessed a different, but related construct to a comparison between combustion and nicotine (or all other sources) to smoking-related harm, we might expect single items assessing the appropriate comparisons to be better predictors.

The difficulty many people experience in understanding risk in quantitative terms is well known to specialists in risk perception and decision psychology, as is the dominance of affect (emotion) over reflection (cognition) in decisions about taking or avoiding risks [29–33]. Our findings further confirm that people have difficulty making probability estimates, and relative measures (more or less of one or the other, e.g., the NVP relative harm measure) were more predictive. More thinking is required on how to help people with these limitations in their thinking.

The four component causes (entered as a block) had additional predictive associations for quit-related outcomes as well as the NVP measures, providing evidence for the generalized concern model of motivation as, at least, having independent influences. However, the two most consistently having an effect were ‘Tobacco’ (unburned) and ‘Additives’, suggesting misperceptions may drive increased interest in quitting (i.e., overestimating risk of these components was associated with stronger plans to quit). The implications of the associations need to be carefully considered. From the perspective of those who believe the ends justify the means, being misinformed about the harm resulting from nicotine, additives or unburned tobacco is advantageous if it motivates complete abstinence (the identified public health goal) regardless of the individual smoker’s values and priorities. However, underestimating the harm from combustion products was also associated with reduced quit-related interest. Correcting this misunderstanding may thus increase interest in quitting but should also lead to a reduction in erroneous beliefs about additives and unburned tobacco. The net effect on motivation is unknown, so it is possible that there would be no net benefit of the misperceptions if smokers understood the dominant role of combustion.

An alternative perspective, one more consistent with the idea that health promotion should enable people to increase control over their health [34], depends on increased understanding of relevant information. In this case, helping people to understand how some other nicotine-containing products are likely far less harmful than combusted tobacco. Currently, smokers’ understandings of the harms of smoking and the causes of those harms appear to be held as compartmentalized facts that are not used in analytic/deliberative thinking. This failure to integrate beliefs into a coherent understanding may be a key factor in persistence of false beliefs. Evidence suggests that people commonly fail to integrate information unless pushed, and may resist it when it conflicts arise with pre-existing beliefs, yet they readily integrate information that supports their behavior-congruent beliefs [35,36]. The task of coming to a coherent understanding is made even harder by the existence of a range of misleading and sometimes simply wrong communications about tobacco and nicotine products which fail to be corrected or put into a coherent perspective (eg that EVALI might have been caused by vaping nicotine; and emotive concerns about nicotine use, particularly among youth). While difficult, the task is achievable. When prompted, most people can make links between information and draw appropriate conclusions, ones they do not make unassisted [18,36,37]. This challenge of integrating knowledge has long been recognized in education research. A core educational goal is to achieve practical knowledge; that is, understanding and critically evaluating information such that it can inform choices [38,39]. Health educators should aim to do this for smoking, but to do so may require finding other ways to reduce cognitive dissonance and to find more effective ways of dealing with unhelpful public communications.

Some smokers want to continue to smoke despite knowing the risk [40] and others are unable to manage the transition costs (e.g., withdrawal) we need to accept that for them regularly considering the harms of smoking just makes them feel bad. That is, they reduce cognitive dissonance by not thinking about the harms they are potentially doing to themselves (ie take dissonance-reducing actions). A realization that their desire to smoke is largely due to nicotine, not the more harmful smoke, may be a more productive pathway to actively resolve their dilemma: that non-smoked nicotine products might be acceptable alternatives to smoking, providing either a replacement for what they want from smoking or a pathway to overcome their current impotence about acting. As harm reduced products become more available and perhaps better substitutes for smoking, encouraging smokers to understand the role nicotine plays in their desire to smoke may play a more critical role in efforts to eliminate tobacco smoking [41,42].

### Limitations

This study has several limitations. Firstly, we presented the four component cause items in the same order, beginning with Nicotine. This may have oriented participants toward addiction or dependence as a form of harm and thus impacted the level of concern about nicotine, relative to the other sources, so caution is required in interpreting absolute levels of beliefs. Second, while the ranges we used for probabilities and the descriptive labels are consistent with the evidence, in some cases they may have been too broad, and the “correct” ranges could have been restricted further. However, this would have reduced the already very small number of smokers with correct responses. Third, we did not ask about all possible sources of harm from smoking. Some respondents may have taken this into consideration. However, if they did, it should have led to more reports that added to less than unity, a pattern which was rare. Fourth, causality cannot be assumed with regards to whether the observed associations are attributable to an integrated understanding of smoking harms. Finally, testing whether more accurate understanding actually leads to more appropriate action requires longitudinal studies.

## Conclusions

Most smokers lack conceptual coherence about smoking harms, with many either not knowing the relative harm that combustion, nicotine, additives, and unburnt tobacco cause or reporting inconsistent responses. Health educators should be aware that many smokers have inadequate knowledge to make informed decisions, and do not automatically make logical integrating links between information. Empowering smokers to make sensible choices will require supporting them to develop the necessary understanding through public health messaging of the relative harms of combustion in relation to other sources of harm from smoking tobacco. This may also help them recognize and reject the misleading messages to which they are often subjected.

## Supporting information

S1 Table Key measures of harm perception. (DOCX)

S2 Table Effect of estimation of combustion harms on plan to quit smoking and want to quit smoking. (DOCX)
