# Supplementary material for: Associations between smokers’ knowledge of causes of smoking harm and related beliefs and behaviors: Findings from the International Tobacco Control (ITC) Four Country Smoking and Vaping Survey
Source: PLoS One. 2023 Oct 24;18(10):e0292856. doi: 10.1371/journal.pone.0292856 (PMC10597481; doi:10.1371/journal.pone.0292856)
Supplement: S1 Table — (DOCX) [file pone.0292856.s001.docx]

**S1 Table: Key measures of harm perception**

| **Variable/Question** | **Response options** | **Results table** |
| --- | --- | --- |
| **Component causes of harm** |  |  |
| *How much of the disease caused by cigarette smoking comes from the following?* |  | Table 2 as DV  Table 4 as IV |
| Combustion in tobacco | 1 None or very little,2 some but less than half, 3 around half, 4 more than half, 5 all or nearly all | Table 2 (“correct” = response 4 or 5)  Table 4 as IV |
| Nicotine | 1 None or very little,2 some but less than half, 3 around half, 4 more than half, 5 all or nearly all | Table 2 (“correct” = response 1 or 2)  Table 4 as IV |
| Other substances in unburnt tobacco | 1 None or very little,2 some but less than half, 3 around half, 4 more than half, 5 all or nearly all | Table 2 (“correct” = response 1,2 or 3)  Table 4 as IV |
| Tobacco additives | 1 None or very little,2 some but less than half, 3 around half, 4 more than half, 5 all or nearly all | 2 (“correct” = response 1 or 2)  Table 4 as IV |
| **Relative concern** |  |  |
| Computed measure derived from the “Nicotine and Combustion” component questions | 1, combustion products rated two or more points higher than nicotine on the 5-point response scales (Combustion most harm); 2, combustion rated one point higher than nicotine (Combustion just); 3, combustion and nicotine rated equally (non-discriminating); 4, nicotine rated higher than combustion (Nicotine most); and 5, ‘Don’t know’: either to both , or Don’t know to nicotine and “half or less for “Combustion’s” role as half or more of the harm was not “accounted for”. | Table 3 as DV  Table 4 as IV |
| **Relative harm** |  |  |
| *Compared to smoking cigarettes, how harmful do you think vaping (using e-cigarettes) is?”* | Much less; Somewhat less: Equally; somewhat more; Much more; don’t know | Table 3 as IV |
