# Supplementary material for: Associations between smokers’ knowledge of causes of smoking harm and related beliefs and behaviors: Findings from the International Tobacco Control (ITC) Four Country Smoking and Vaping Survey
Source: PLoS One. 2023 Oct 24;18(10):e0292856. doi: 10.1371/journal.pone.0292856 (PMC10597481; doi:10.1371/journal.pone.0292856)
Supplement: S2 Table — (DOCX) [file pone.0292856.s002.docx]

**S2 Table**. **Effect of estimation of combustion harms on plan to quit smoking and want to quit smoking**

|  | **Estimation of harm caused by Combustion** | | | | | |  |  |
| --- | --- | --- | --- | --- | --- | --- | --- | --- |
| **Outcome *Component cause*** | **None or  very little** | **Some but less than half** | **Around**  **half** | **More than half** | **All or nearly all of it** | **Don't know** | **Chi** | **p-value** |
|  | N=226 | N=1,462 | N=1,648 | N=1,828 | N=1,531 | N=1,816 |  |  |
| **Plan to quit smoking** | | | | | | |  |  |
| No immediate plan | 47.8 | 33.4 | 27.8 | 25.4 | 25.7 | 47.4 | 338.8 | <.001 |
| Beyond 6 months | 26.1 | 33.7 | 33.9 | 36.2 | 31.4 | 27.6 |  |  |
| Between 1-6 months | 16.8 | 24.7 | 25.8 | 26.0 | 28.3 | 18.0 |  |  |
| Within 1 month | 9.3 | 8.2 | 12.5 | 12.3 | 14.6 | 7.0 |  |  |
| **Want to quit smoking** | |  |  |  |  |  |  |  |
| No desire | 30.5 | 13.9 | 11.7 | 8.2 | 7.6 | 19.2 | 395.6 | <.001 |
| A little | 23.0 | 23.5 | 21.2 | 18.0 | 13.0 | 17.6 |  |  |
| Somewhat | 24.8 | 35.5 | 32.8 | 34.6 | 30.6 | 34.4 |  |  |
| A lot | 21.7 | 27.2 | 34.3 | 39.2 | 48.9 | 28.8 |  |  |
| Column percentage shown | |  |  |  |  |  |  |  |
